# Cost-Effective Fitting Model for Indoor Positioning Systems Based on Bluetooth Low Energy

**DOI:** 10.3390/s22166007

**Published:** 2022-08-11

**Authors:** Sheng-Cheng Yeh, Chia-Hui Wang, Chaur-Heh Hsieh, Yih-Shyh Chiou, Tsung-Pao Cheng

**Affiliations:** 1 Department of Information and Telecommunication Engineering, Ming Chuan University, No. 5 Der-Ming Rd., Gwei Shan District, Taoyuan City 333, Taiwan; 2 Department of Computer Science and Information Engineering, Ming Chuan University, No. 5 Der-Ming Rd., Gwei Shan District, Taoyuan City 333, Taiwan; 3 College of Artificial Intelligence, Yango University, No. 99 Denglong Road, Mawei District, Fuzhou 350015, China; 4 Department of Electronic Engineering, Chung Yuan Christian University, No. 200 Zhongbei Rd., Zhongli District, Taoyuan City 320, Taiwan; 5 Department of Computer and Communication Engineering, Ming Chuan University, No. 5 Der-Ming Rd., Gwei Shan District, Taoyuan City 333, Taiwan

**Keywords:** indoor positioning, RSS, BLE, fitting model

## Abstract

Bluetooth Low Energy (BLE) is a positioning technology that is commonly used in indoor positioning systems (IPS) such as shopping malls or underground parking lots, because of its low power consumption and the low cost of Bluetooth devices. It also maintains high positioning accuracy. Since the cost of BLE itself is low, it has now been used in larger environments such as parking lots or shopping malls for a long time. However, it is necessary to configure a large number of devices in the environment to obtain accurate positioning results. The most accurate method of using signal strength for positioning is the signal pattern-matching method. The positioning result is compared through a database with the overheads of time and labor costs, since the amount of data will be proportional to the size of the environment for BLE-IPS. A planar model that conforms to the signal strength in the environment was generated, wherein the database comparison method is replaced by an equation solution, to improve various costs but diminish the positioning accuracy. In this paper, we propose to further replace the planar model with a cost-effective fitting model to both save costs and improve positioning accuracy. The experimental results demonstrate that this model can effectively reduce the average positioning error in distance by 31%.

## 1. Introduction

With the advances in smart phones, mobile devices and wireless networks, applications and developments in Location-Based Services (LBS) have steadily progressed, with increasing accuracy in positioning. Depending on the situation, positioning services can extend to different functions, including weather forecasts, bus information, and even games. It can be observed that people’s lives are inseparable from positioning services. Global Positioning System (GPS) is an increasingly mature positioning service [1] which uses triangulation for positioning. However, effective satellite signals cannot be obtained where there are obstacles, which makes it impossible to accurately apply GPS in indoor spaces with many obstacles. In addition to obstacles, signal interference and multipath propagation are also challenges faced in indoor environments. Hence, indoor positioning technology (IPS)-related research is constantly innovating, including Wi-Fi, Bluetooth Low Energy (BLE), ZigBee and infrared technology. Among them, BLE is a positioning technology that is more commonly used in shopping malls or underground parking lots because of its relatively low power consumption and the low cost of Bluetooth devices, whilst maintaining relatively high positioning accuracy. Although the cost of BLE itself is relatively low, the positioning method used today requires a large number of devices to be deployed in large spaces such as parking lots or shopping malls to obtain accurate positioning results. The equipment cost is proportional to the size of the environment.

As early as 2000, a Microsoft team reported on an indoor positioning system called Radio Detection and Ranging (RADAR) [2], which was based on the signal strength comparisons for positioning. Subsequent studies also used this concept to perform indoor positioning through Wi-Fi wireless base stations distributed throughout public places such as transportation stations, schools, and department stores. This positioning method required collection of the signal strength characteristics of the reference points in the environment, establishment of a database of this data, and then location of the signal strength characteristics between the reference points. However, this method required considerable time and manpower, both in the establishment of the database and the comparative analysis of the positioning; moreover, these costs were proportional to the size of the environment. In order to decrease labor and time costs, a method of replacing the signal feature comparison with a planar model was proposed. The formula was based on a model that matches the signal strength of reference points in the environment, instead of the signal strength feature comparison method [3]. This method effectively improved the defects of the comparison method, but the positioning accuracy was impacted.

The purpose of this current research is to perform accurate indoor positioning while reducing associated costs. Our IPS system is primarily divided into two stages of offline deployment and online positioning. The coordinates and signal strengths of reference points in the environment are collected in offline stage. Then, the fitting model and its equation can be generated through a toolbox of mathematical simulation and model design. In the online stage, user can conveniently use a smartphone to obtain the IPS location from the strength of received signal through the fitting model equation generated in the offline stage. Thus, the merit of this study uses BLE to perform IPS includes the following:

- Its relatively low unit cost, as the signaling device, and bases its positioning method on the planar model equation in order to save labor and time costs.
- A model that more closely matches the signal strength of the reference points in the environment can be established to replace the original planar model.
- The advantages of collecting less signal strength data from reference points is retained in order to control the equipment cost and manpower/time expenses for establishment of the comparison database, while improving the positioning accuracy of the planar model.

## 2. Related Works

Whether it is Wi-Fi, BLE, RFID, or others, distance measurement and positioning are achieved through the path loss characteristics of radio waves; that is, signal strength gradually decreases with increased distance. However, in indoor environments, signals are easily affected by multipath propagation, making IPS research challenging. Nowadays, there are many studies related to positioning measurement methods, which can be divided into three categories, namely time-based methods, angle-based methods, and received signal strength-based methods. These three methods were explained in [4]. The popular received signal strength-based methods and other positioning systems are explained below.

### 2.1. Received Signal Strength-Based Methods

Most of the positioning technology developed using received signal strength (RSS) technology is based on the attenuation value of signal strength between the transmitter and the receiver to measure the distance. The prediction model [2] for calculating distance based on signal transmission is shown in Formula (1).

(1) $$P\left(d\right)=P\left(d_{0}\right)-10n\log\left(\frac{d}{d_{0}}\right)-\left\{\begin{array}{c} nW\times WAF\;(nW<C)\\ C\times WAF\;\;\;\left(nW\geq C\right) \end{array}\right.$$

*P*(*d*) is the signal strength value of the user’s location; *d* is the distance from the wireless BS; $P\left(d_{0}\right)$ is the signal strength of the reference point; $d_{0}$ is the distance between the reference point and the wireless BS; *n* is parameter related to the signal strength attenuation due to the distance; *nW* is the number of walls; *C* is the maximum allowable number of walls; *WAF* is the wall attenuation factor; *WAF* will vary depending on the wall material.

This model was proposed as RADAR by Microsoft in order to reduce the cost of the offline stage in IPS. It requires considerable time and manpower to collect the signal strength characteristics of each reference point, and these costs will be proportional to the size of the environment. Microsoft uses radio signals from indoor Wi-Fi wireless base stations for IPS using RADAR positioning technology, as published by IEEE INFOCOM in 2000. The RADAR system is the RSS positioning method based on signal strength, which can be divided into two stages, namely the offline stage and the online stage [2].

**Figure 1:**
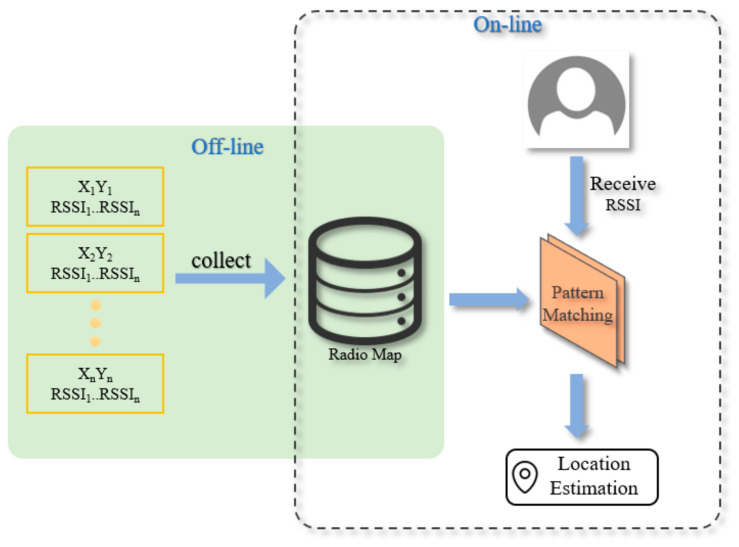
RADAR Positioning Technology Flowchart [2].

In the offline stage, the RSSI of each reference point (i.e., access point, AP) is collected multiple times. Because the signal strength received for each reference point is different, the characteristics of the individual signal patterns can be obtained and sorted for the database. In the subsequent online stage, the signal strength received through the current location is compared with the signal pattern database created in the offline stage to derive the user’s position based on a comparison result from the signal pattern matching. Thus, this is called a signal pattern-matching method as shown in Figure 1.

The RSSI received during the online stage is compared with the database created in the offline stage to obtain the user’s position. The Euclidean distance Formula (2) is used for calculation with the RSSI obtained online and all RSSIs in the database, where *X* is the RSSI of the user’s location; *Y* is the RSSI of the reference point in the database; *n* is the number of base stations; and *d*(*x*) is the comparison between the user and the database reference point after calculation. As a result, the number of reference points in the database is the number of *d*(*x*) solutions, and from all *d*(*x*), the reference point closest to the user can be calculated.

(2) $$d\left(x\right)=\sqrt{{\left(X_{1}-Y_{1}\right)}^{2}+{\left(X_{2}-Y_{2}\right)}^{2}+\ldots +{\left(X_{n}-Y_{n}\right)}^{2}}$$

The signal pattern-matching method is currently a common and highly accurate method for IPS. However, because the positioning method continuously compares the data in the database, the amount of calculation is complex. When the positioning environment is larger, more signal pattern data are required for comparison; thus, the calculation complexity also increases. Therefore, reducing the computational complexity has become a problem that needs to be considered to effectively improve the signal pattern-matching IPS, and the use of models to replace the comparison method has become an effective means to reduce the computational complexity.

**Figure 2:**
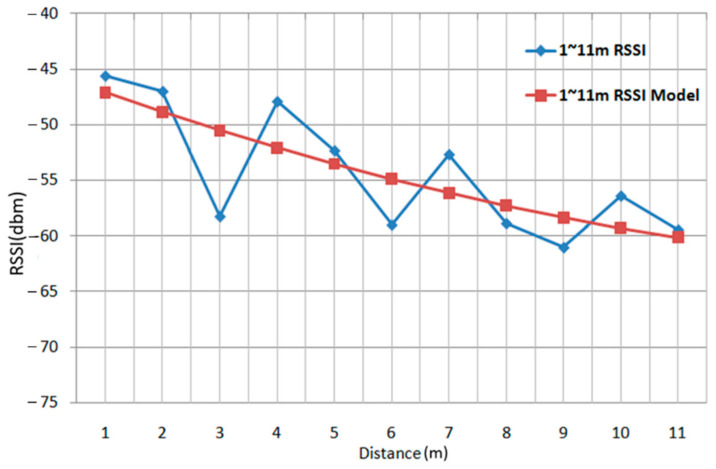
Graph of Relationship between Signal Intensity and Distance [6].

Numerous studies have explored the relationship between signal strength and the distance between wireless base stations and devices [5,6,7], and have found the relationship to be inversely proportional. However, the curve in the relationships fluctuates up and down without the rule of linearity. In order to reduce the computational complexity, an equation is used instead of signal pattern comparison; the linear model obtained from the equation is used to replace the signal strength curve, as shown in Figure 2.

**Figure 3:**
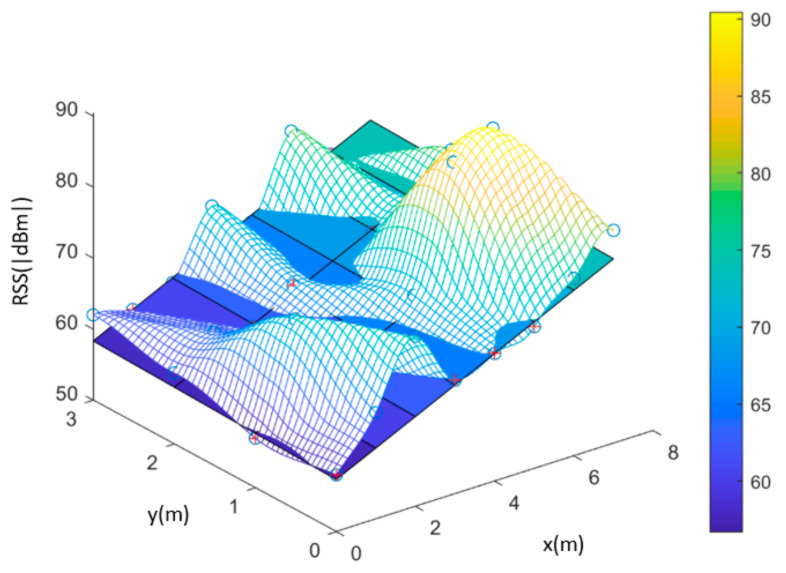
Actual Signal Intensity Distribution and the Planar Model.

Some studies have suggested that signal strength could first be collected separately in individual spaces, such as the bedroom, the living room or the kitchen. Then, the “signal strength surface” of the space can be obtained for each base station. Through the signal strength surface, the least-squares method [8] can be used to calculate the plane, and this plane can be used for the positioning model. A planar model can be obtained for each space corresponding to each wireless base. By just detecting which space the user is in, the planar model of the space can be used to locate the user’s position. When the strength at a specific location is known, the location can be determined through solving the simultaneous equations by simply inputting the strength into the two planar models. This replaces the massive data comparison method, achieving the goal of reducing calculation complexity [3]. Figure 3 is an example of actual signal strength distribution and the planar model.

### 2.2. Other Positioning Systems

The ZigBee positioning system uses the triangulation method for positioning; it does not use signal transmission and reception time difference for distance conversion, rather it uses the signal and distance relationship model to convert the signal strength into distance. Equation (3) presents the ZigBee model based on the relationship between signal strength and distance [9].

(3) $$\mathrm{RSSI}=A-10n\log_{10}\left(\frac{d}{d_{0}}\right)$$

where RSSI is the signal strength at the receiver; *d* is the distance between the receiver and the transmitter; $d_{0}$ is the distance in measurement units; *n* is the signal attenuation value; and *A* is the signal strength per unit distance. Using this model, the relationship between ZigBee signal strength and distance can be obtained, and triangulation can be used for positioning. However, because there are many obstacles in most rooms, shadowing effects, or signal diffraction and reflection easily occur. The resulting multipath effect leads to error between the calculated transmission distance and the actual distance, so the calculated position is relatively inaccurate.

Radio frequency identification (RFID) positioning technology detects a tag through the RFID reader, and knows the range of the RFID reader where the tag is located. However, the range of RFID is quite limited. The ultra-high frequency (UHF) RFID tag can only reach a transmission distance of 5 m. This equipment is mostly used to determine whether an object is present at or passes through a preset position; it is impossible to detect exactly where the object is. RFID is not suitable for indoor positioning due to its small transmission range.

The iBeacon [10], a BLE device officially launched by Apple, is based on Bluetooth-based positioning technology. Compared with other technologies such as Wi-Fi, due to its small size, low power consumption and low cost, it is more suitable for applications in various commercial environments and indoor venues. Nowadays, most Bluetooth positioning technologies use multiple iBeacons installed inside as base stations to transmit signal strength, that is, RSS. Similar to Wi-Fi, different transmission distances will have different degrees of attenuation. After receiving the signal strength, the receiving port can calculate the distance between the receiving port and each iBeacon, and then apply a triangulation method to obtain the location of a mobile phone in the area. However, the positioning accuracy and stability of BLE is still affected by human body shadowing and multipath interference.

Studies in [11,12,13,14,15] are related to Bluetooth indoor positioning technology in recent years. The study in [11] added the Bluetooth signal strength to the weighting, and used a signal pattern comparison method for positioning. Ref. [12] used iBeacon combined with polynomial regression model (PRM), fingerprinting (FP) and extended Kalman filter technology for indoor positioning. Using FP and PRM to estimate the position of the target and the distance between the target and iBeacon, respectively, algorithm achieved an accuracy of less than 2.56 m when the beacons were densely deployed (one iBeacon per nine meters). Ref. [13] combined Wi-Fi and BLE technologies with radio fingerprinting to allow users to create radio maps and update them continuously. Ref. [14] used signal-to-noise ratio (SNR) and Bluetooth signal strength to perform positioning calculations through a machine learning service provided by Amazon Web Services (AWS). Ref. [15] proposed a particle filter-based indoor positioning system to localize tags that can broadcast BLE beacon messages. To reduce fluctuations of RSSI data, they design a Kalman filter to smooth those data. Based on the smoothed RSSI data, they propose a particle filter to conduct IPS tasks. Though the above methods are all aimed at improving positioning accuracy, they also increase the burden of computational complexity. Comparisons of characteristics in various positioning technologies are summarized in Table 1.

## 3. System Architecture and Proposed Method

### 3.1. System Architecture

**Figure 4:**
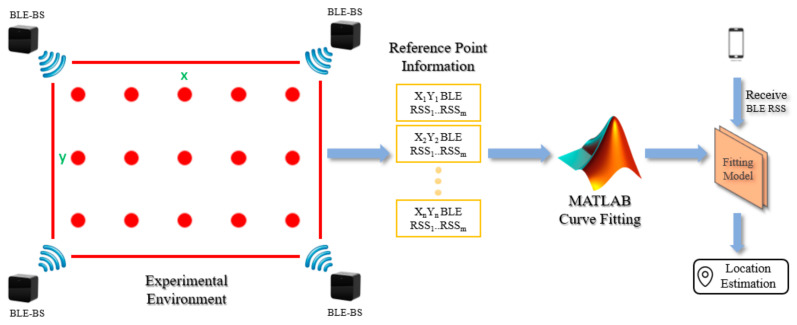
Schematic Diagram of System Architecture.

The research architecture of this study is shown in Figure 4 as a schematic diagram. The Bluetooth signal is selected to set up the experimental environment, and, due to its low cost, low power consumption and convenient features, a mobile phone is used as a signal receiving device. Four Bluetooth base stations are set up to collect the X and Y coordinate values of the reference points in the environment and the signal strength of each base station through the mobile phone; then the data obtained are used to generate a fitting model through MATLAB to carry out positioning [17]. The larger the displayed value of signal strength, the stronger the signal strength. Moreover, this research selects the Bluetooth-strength-transmitting device provided in HTC VIVE as a base station because the system itself includes this device and it can provide a stable Bluetooth signal [18].

**Figure 5:**
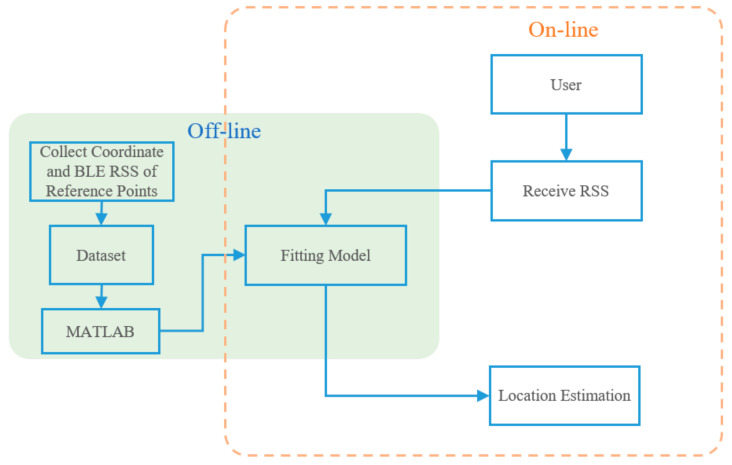
System Flowchart for This Study.

The system is primarily divided into two stages from establishment to positioning, namely the offline stage and the online stage. In the offline stage, the coordinates and signal strength of each reference point in the environment are collected; then the fitting model and its equation are generated through MATLAB. In the online phase, when in the positioning environment, the user may conveniently use a mobile phone to obtain the Bluetooth signal strength in the environment, and then the location of the user obtained through the fitting model equation generated when offline, as shown in Figure 5.

Based on the above system architecture and flowchart, our research method for the cost-effective fitting for IPS, as described in following Section 3.3, can reduce both labor cost and latency while comparing with traditional signal pattern matching’s complexity and time cost.

### 3.2. Experimental Environment

**Figure 6:**
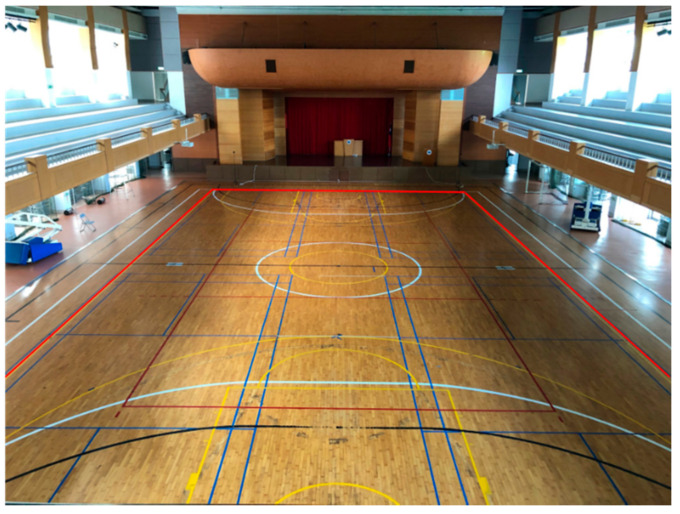
Experimental Environment–Basketball Court.

In order to verify whether the fitting model can be applied to an indoor environment, the experimental environment of this research is set as the size of a standard basketball court (the one employed in this study is in the second gymnasium on the Taoyuan Campus of Ming Chuan University), as shown in Figure 6. Four BLE wireless base stations are set up in the four corners of the environment; the distance between each reference point is two meters. A smart phone is used to collect 20 data points of signal strength for each Bluetooth base station at each reference point. These data are sorted, and the average of the middle 10 signal readings is set as the signal characteristic at that point to avoid the impact of interference on the signal. Following this, the signal characteristics of each reference point are collected and MATLAB generates a fitting model in accordance with these data.

### 3.3. Research Method

This section describes the methods used in this research. In order to compare the proposed fitting model and the planar model, the models are generated in the same way and the same method is used for positioning determination.

#### 3.3.1. Model Production

In this study, the models are produced mainly by generating signal characteristics collected from the reference points in the environment with MATLAB tools. Three steps explain the process of model generation, as follows:

**Step 1**. Data Collection and Collation

**Figure 7:**
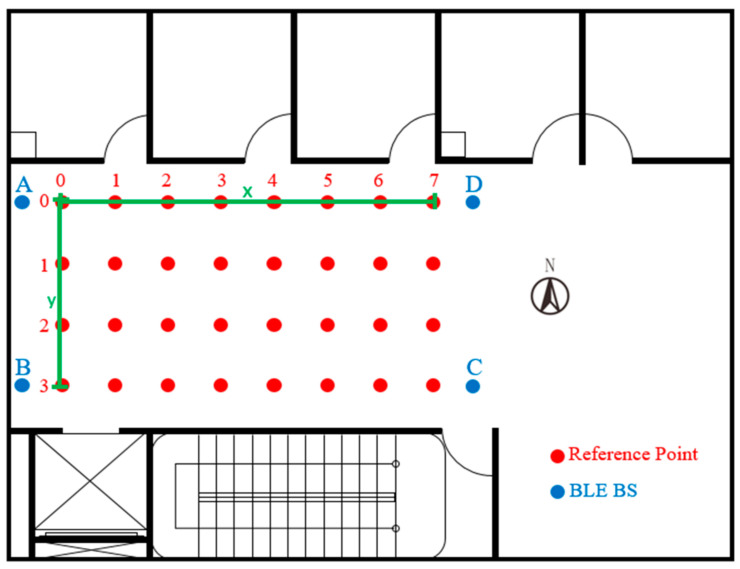
Schematic Diagram of Planned Reference Points in the Environment.

**Figure 8:**
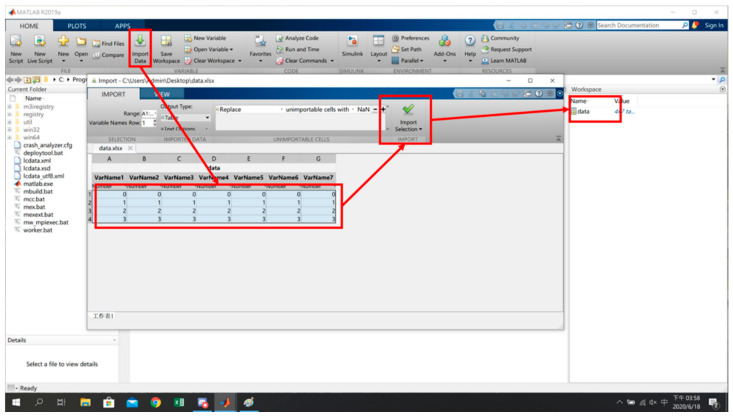
Input of Reference Point Data into MATLAB [17].

As an example, in Figure 7, taking the long side as the *x*-axis, the short side as the *y*-axis, and the distance (meters) as the coordinate values of the planned reference points in the environment, x, y and signal characteristics of all reference points are sorted out. The sorted data are imported into MATLAB (as shown in Figure 8) by selecting Import Data and the target Excel file to open the import interface. After selecting the required data range, the data imported into MATLAB are available for subsequent actions generated by the models.

**Step 2**. MATLAB Fitting Model Tools

**Figure 9:**
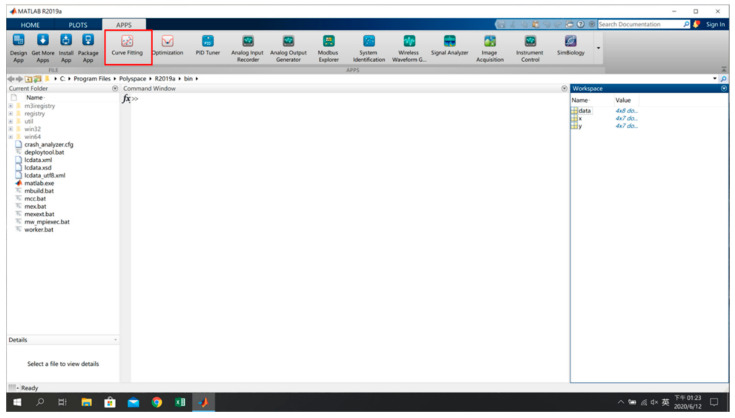
Curve Fitting Tool.

Generally speaking, a mathematical model established by curve fitting is single-input single-output (SISO), so its characteristics can be represented by a curve. The characteristics of the two imported mathematical models can be represented by a curved surface, a type of problem called surface fitting. Whether curve fitting or surface fitting, in data analysis, they are both referred to as regression analysis or data fitting. Regression analysis is closely related to the mathematical model used. If the model used is a linear model, this type of problem is called linear regression; if a nonlinear model is used, it is called nonlinear regression. The fitting model to be produced in this research is a nonlinear regression, which is a more difficult problem than linear regression, because the best solution cannot be found at one try, one cannot guarantee that the best solution can be found, so must try various nonlinear methods of transformation to find the best fit, and related mathematical properties are often not clear. Due to the above reasons, this research uses the curve fitting tool in MATLAB, as shown in Figure 9, which provides the function of fitting curves and surfaces to the data, with linear and nonlinear model libraries for regression analysis, and allows for specification of one’s own custom equations.

**Step 3**. Custom Equation

**Figure 10:**
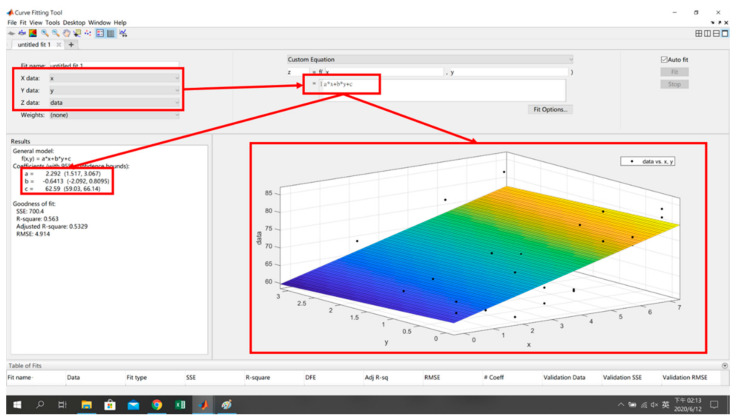
Planar Model and Equation Generation.

**Figure 11:**
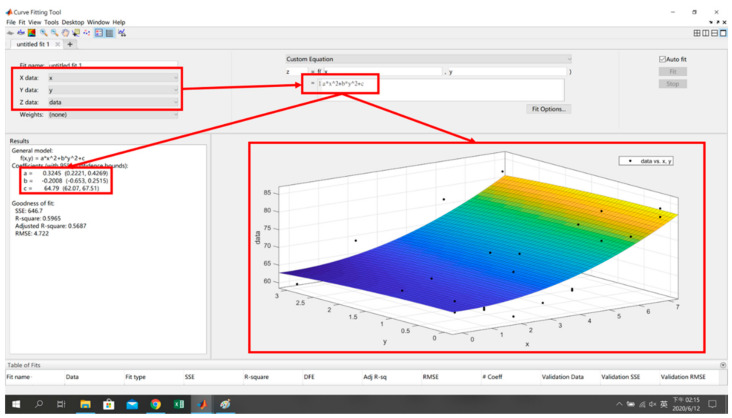
Fitting Model and Equation Generation.

The *x*, *y* and signal characteristics are sorted out in Step 1, input into the curve fitting tool in Step 2, and then the custom equation method is used to formulate the plane (4) and the surface (5) to obtain the fitting model and its equations, where *s* is the signal strength of the reference point, and *x* and *y* are the coordinates of the reference point, as shown in Figure 10 and Figure 11.

(4) $$s=f\left(x,y\right)=ax+by+c$$

(5) $$s=f\left(x,y\right)=ax^{2}+by^{2}+c$$

#### 3.3.2. Fitting Model and Equation Generation

**Figure 12:**
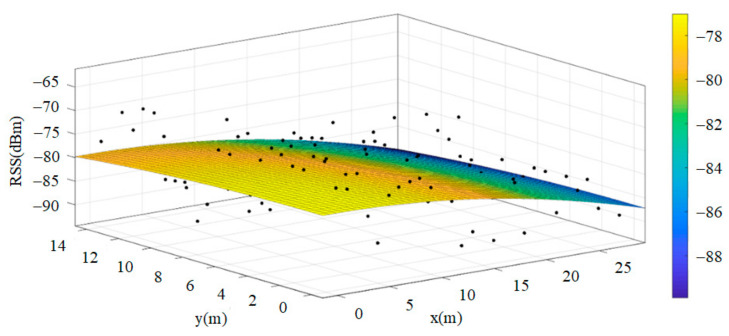
Fitting Model Generated for Base Station A.

**Figure 13:**
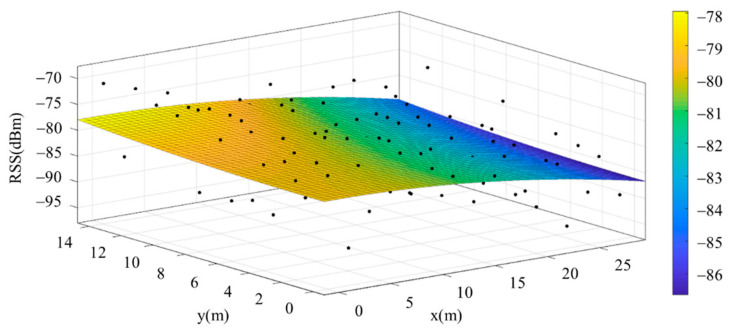
Fitting Model Generated for Base Station B.

**Figure 14:**
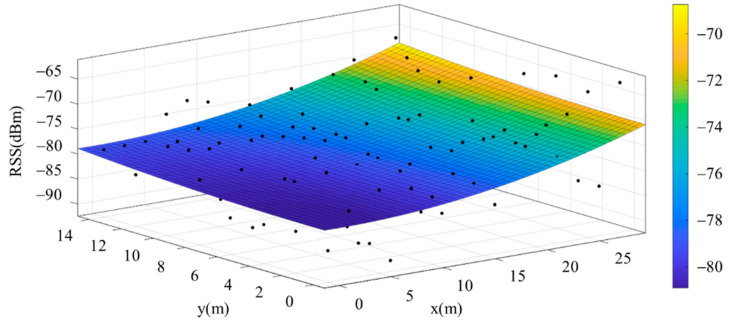
Fitting Model Generated for Base Station C.

**Figure 15:**
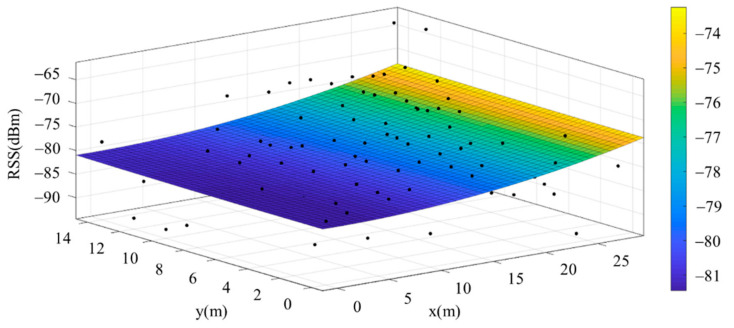
Fitting Model Generated for Base Station D.

The reference points in the environment all have their own coordinates, but different fitting models are generated for different base stations. Figure 12, Figure 13, Figure 14 and Figure 15 are based on the environment of the gymnasium where the basketball court employed in this research is located. The individual models of the base stations, and their equations are shown in Table 2.

**Figure 16:**
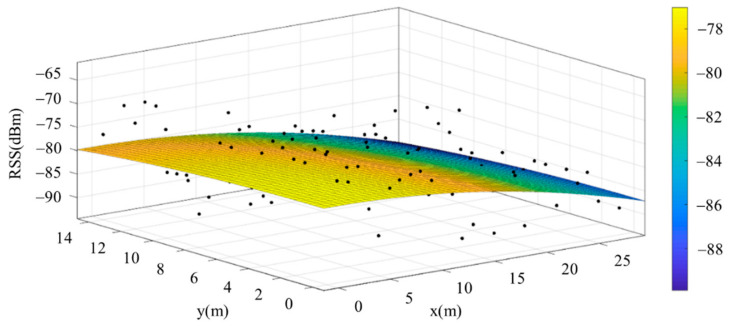
Fitting Model for Same Base Station Using Data Collected Facing East.

**Figure 17:**
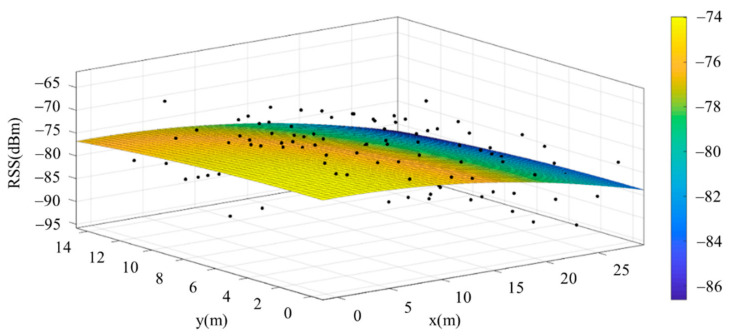
Fitting Model for Same Base Station Using Data Collected Facing West.

**Figure 18:**
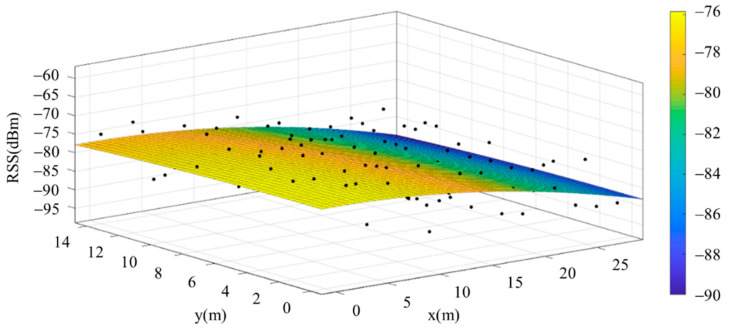
Fitting Model for Same Base Station Using Data Collected Facing South.

**Figure 19:**
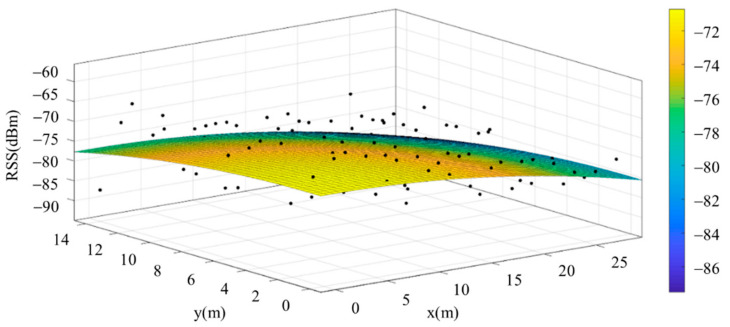
Fitting Model for Same Base Station Using Data Collected Facing North.

In addition to the different fitting models generated by different base stations, in order to avoid the human body shadow of the data collector affecting the signal characteristics during the process of collecting signal strength, this study collected signal characteristic data in the experimental environment from all four directions: north, south, east and west. Figure 16, Figure 17, Figure 18 and Figure 19 are the individual fitting models generated for the same base station with respect to data collected from the four different directions in the experimental environment. The equations are shown in Table 3.

With four base stations and four directions relative to the environment, 16 sets of fitting models and equations are obtained. Thereafter, by solving the equations, the user’s position can be determined.

#### 3.3.3. Equation Solutions and Positioning Determinations

By solving the simultaneous model equations obtained, a solution can be obtained indicating the likely position of the user. The planar model determination can be solved through Equations (6)–(9).

(6) $$s_{1}=f\left(x,y\right)=a_{1}x+b_{1}y+c_{1}$$

(7) $$s_{2}=f\left(x,y\right)=a_{2}x+b_{2}y+c_{2}$$

(8) $$x=\left(b_{1}c_{2}-b_{2}c_{1}-b_{1}s_{2}+b_{2}s_{1}\right)/\left(a_{1}b_{2}-a_{2}b_{1}\right)$$

(9) $$y=-\left(a_{1}c_{2}-a_{2}c_{1}-a_{1}s_{2}+a_{2}s_{1}\right)/\left(a_{1}b_{2}-a_{2}b_{1}\right)$$

Compared with the planar model, the solution for the surface model is more complicated, involving Equations (10)–(15). Due to the squared relationship, four sets of solutions are generated; among them are some that are unrealistic solutions. After eliminating the unrealistic ones, the user’s possible position can be determined.

(10) $$s_{1}=f\left(x,y\right)=a_{1}x^{2}+b_{1}y^{2}+c_{1}$$

(11) $$s_{2}=f\left(x,y\right)=a_{2}x^{2}+b_{2}y^{2}+c_{2}$$

(12) $$\left\{\begin{array}{c} x=\sqrt{\left(b_{1}c_{2}-b_{2}c_{1}-b_{1}s_{2}+b_{2}s_{1}\right)/\left(a_{1}b_{2}-a_{2}b_{1}\right)}\\ y=\sqrt{\left(-\left(a_{1}c_{2}-a_{2}c_{1}-a_{1}s_{2}+a_{2}s_{1}\right)/\left(a_{1}b_{2}-a_{2}b_{1}\right)\right)} \end{array}\right.$$

(13) $$\left\{\begin{array}{c} x=\sqrt{-\left(\left(b_{1}c_{2}-b_{2}c_{1}-b_{1}s_{2}+b_{2}s_{1}\right)/\left(a_{1}b_{2}-a_{2}b_{1}\right)\right)}\\ y=\sqrt{\left(-\left(a_{1}c_{2}-a_{2}c_{1}-a_{1}s_{2}+a_{2}s_{1}\right)/\left(a_{1}b_{2}-a_{2}b_{1}\right)\right)} \end{array}\right.$$

(14) $$\left\{\begin{array}{c} x=\sqrt{\left(b_{1}c_{2}-b_{2}c_{1}-b_{1}s_{2}+b_{2}s_{1}\right)/\left(a_{1}b_{2}-a_{2}b_{1}\right)}\\ y=\sqrt{-\left(-\left(a_{1}c_{2}-a_{2}c_{1}-a_{1}s_{2}+a_{2}s_{1}\right)/\left(a_{1}b_{2}-a_{2}b_{1}\right)\right)} \end{array}\right.$$

(15) $$\left\{\begin{array}{c} x=\sqrt{-\left(\left(b_{1}c_{2}-b_{2}c_{1}-b_{1}s_{2}+b_{2}\right)/\left(a_{1}b_{2}-a_{2}\right)\right)}\\ y=\sqrt{-\left(-\left(a_{1}c_{2}-a_{2}c_{1}-a_{1}s_{2}+a_{2}s_{1}\right)/\left(a_{1}b_{2}-a_{2}b_{1}\right)\right)} \end{array}\right.$$

**Figure 20:**
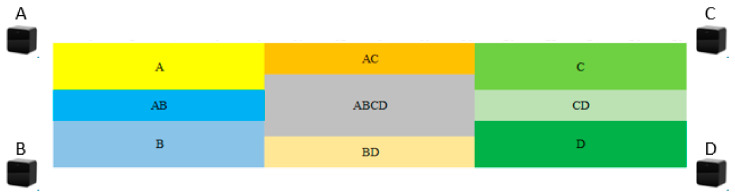
Distribution of Determination Blocks According to the Closest Base Station.

One solution can be obtained for every set of two equations, but considering the directional issue, this research only applies the model equations for the same direction to solve the equations. Therefore, the original 16 sets of model equations yield 120 sets of solutions, and the 4 sets of model equations for the same direction yield 6 sets of solutions, for a total of 24 sets of solutions from the 4 directions. Then, by determining the area where the user is located, taking the experimental environment of the basketball court as an example, Figure 20 shows the environmental area divided into blocks by the base station closest to it, and the user’s position is determined by the strength of the base station signal received by the user. The average distance between the 24 sets of solutions and the four corners of the determined area is calculated, and the smallest value solution is the one that determines the location of the user. However, if the distance of the smallest value is greater than the maximum distance of the determined area, a compensatory method is used. This method determines which one of the four corners of the determined block the user is in based on the signal strength of each base station received by the user.

## 4. Evaluation Results and Analysis

### 4.1. Results Produced by the Models and Their Comparison

With Table 4, Table 5, Table 6 and Table 7 show the planar and fitting models generated for the experimental environment of the basketball court from the four directions of east, south, west and north for each respective base station equation. It can be seen that, compared with the planar model, the fitting model is more aligned with the signal strength collected at each reference point in the experimental environment. Following this, from the error distance and cumulative distribution function (CDF) results, it can also be seen that the closer the model to the environmental data, the better the results that can be obtained.

### 4.2. Signal Pattern Comparison

In addition to the comparison between models, this study also applies the signal pattern comparison method. A Euclidean distance formula, such as (16), is used to calculate the location of the user [19].

(16) $$d\left(x\right)=\sqrt{{\left(S_{A}-S_{A}^{\prime }\right)}^{2}+{\left(S_{B}-S_{B}^{\prime }\right)}^{2}+{\left(S_{C}-S_{C}^{\prime }\right)}^{2}+{\left(S_{D}-S_{D}^{\prime }\right)}^{2}}$$

Among them, $S_{A}^{\prime }$, $S_{B}^{\prime }$, $S_{C}^{\prime }$ and $S_{D}^{\prime }$ are the average of the signal strength received from four directions at each reference point for the four base stations in the offline stage. $S_{A}$, $S_{B}$, $S_{C}$ and $S_{D}$ are the signal strength values from the four base stations currently received by the user. $d\left(x\right)$ is the comparative error between the signal strength received by the user and the signal strength in the offline data. After comparing with all reference points, the corresponding $d\left(x\right)$ of each point can be obtained. The smaller the $d\left(x\right)$ value, the closer the user is to this point, which informs as to the user’s position.

### 4.3. Comparison and Analysis of Both Methods

The model equation solution is different from the signal pattern-matching method. After the signal pattern comparison is compared with all reference points in the environment, the closest match will be regarded as the positioning result. Therefore, the error distance for the correct point is zero. However, as the solutions obtained through the model equations are approximated, they cannot perfectly match the coordinates of the reference point; some slight errors exist. In addition, when the experimental environment expands and the number of base stations is maintained, the average positioning error will increase. Table 8 shows the average positioning error distances obtained for different environment sizes and different methods in the experimental environment of the basketball court. 

Table 9 shows that the fitting model proposed by this research can effectively improve the average positioning error distance ratio of the planar model. From the original data without area determination, it can be seen that the average positioning error distance is effectively improved by 31%. Although it can effectively improve the planar model, there is still a gap with the signal pattern comparison method. Hence, the area determination is added so that the average positioning error distance of the model can be approximated by the signal pattern comparison method. The area determination is based on the current received signal strength from the base station, but the received strength may be affected by multipath interference, etc., which may lead to area determination errors. Therefore, this study tested the complete accuracy of the area determination and compared the results. There is room for improvement in the area determination method.

Although the fitting model is inferior to the signal pattern comparison method for average positioning error distance, the solution of the model equation can indeed effectively reduce the labor and time costs consumed in the offline phase. Table 10 shows the results of the model generated after measuring reference points at 2, 4, and 8 m apart in the same experimental environment. It can be seen from the results that reducing the amount of reference points in the environment has less impact on the average positioning error distance through the model equation solution.

**Figure 21:**
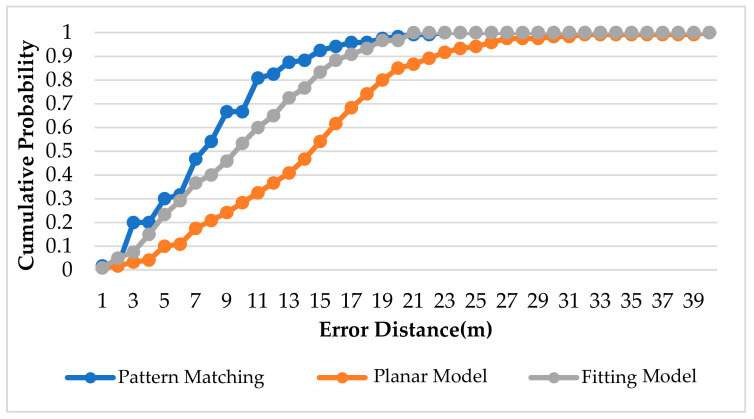
CDF for Original Data in Basketball Court.

**Figure 22:**
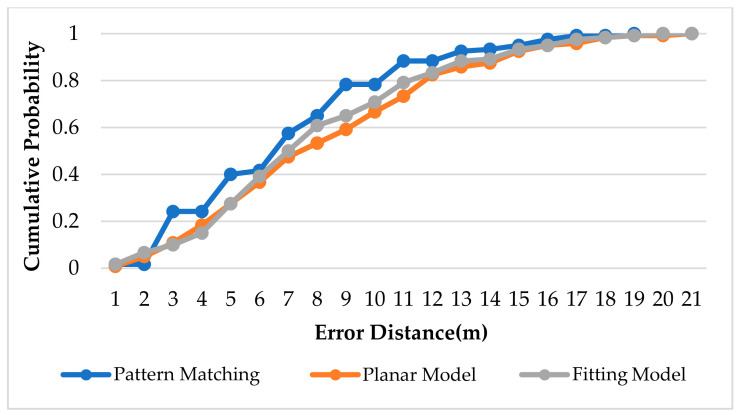
CDF for Area Determination in Basketball Court.

**Figure 23:**
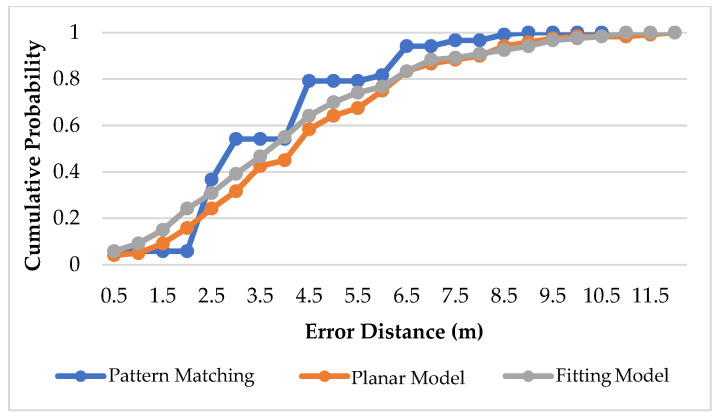
CDF for Optimal Area in Basketball Court.

Figure 21, Figure 22 and Figure 23 show the CDF diagram for the original data for the basketball court, the area determination and the optimal area. It can be seen that these CDF diagrams all show that the fitting model converges faster than the planar model, which confirms that the more the model conforms to the signal strength characteristics of the reference points in the environment, the better the positioning accuracy.

### 4.4. Model Optimization

**Figure 24:**
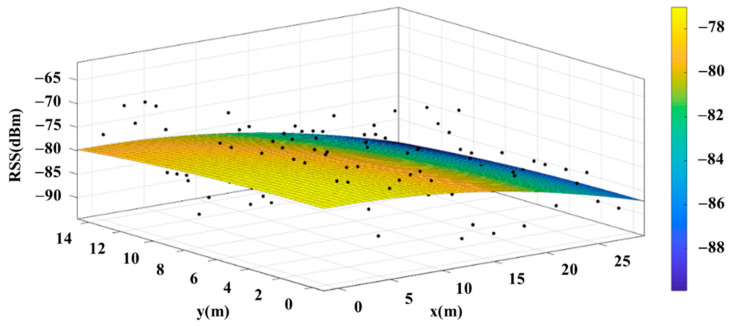
Model Generated Using Simplified Surface Equations.

**Figure 25:**
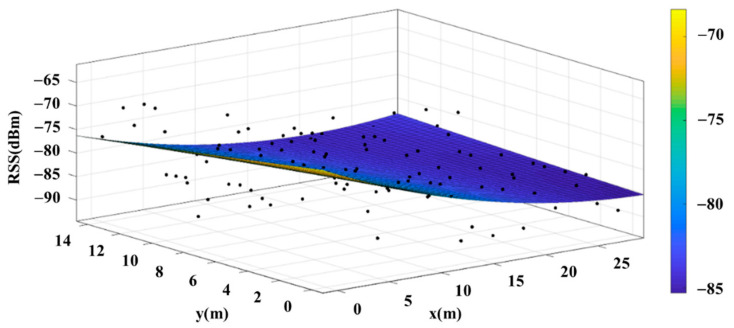
Model Generated Using Surface Normalizing Equations.

The fitting model produced in this study uses simplified surface equations, and the resulting model is shown in Figure 24. For optimization of the model to align it better with the signal strength of each reference point in the environment, a surface normalizing formula must be used (17). The resulting model is shown in Figure 25, but the equations are complicated to solve and positioning cannot be obtained through the equation solutions, so this study gives priority to using simplified surface equations.

(17) $$z=f\left(x,y\right)=ax^{2}+bxy+cy^{2}+dx+ey+f$$

## 5. Conclusions and Future Work

The fitting model proposed in this research is better than the planar model, as it can effectively reduce the average positioning error by up to 31%, and the results after area determination can approximate the signal pattern-matching method. In addition, changing the distance between the reference points has proven that the solution of the model equation can greatly reduce the manpower and time costs of the offline phase. The fitting model proposed in this research successfully reduced various costs and improved the poor positioning accuracy of the planar model.

As for future research direction, the optimization of the model and the determination of which block the user is in can be improved. The optimization of the model, as stated in the research results and analysis, can produce a model that more closely fits the signal strength distribution in the environment with a surface normalization equation, but the disadvantage is that the equation cannot be solved, so the process of obtaining a positioning solution is difficult. As for the determination of the area where the user is, the method used at this time shows that there is still a gap with the optimal situation. 

In the future, we hope to add Landmarks in indoor environment to dynamically calibrate, correct and optimize the fitting models to timely know user’s accurate area, while the changing from types of user’s smartphone and indoor settings such as furniture or office desks in more realistic scenario. For the BLE devices with unstable signals, we believe the moving average can be used to smooth out the noise to find out better fitting model. In addition, sensor-related tools on mobile phones could also be used, such as a gyroscope and an acceleration sensor, through which the user’s location could be known to improve the positioning accuracy. After applying the above two points, the improved method could be tried out in large environments such as an indoor parking lot or multi-story building for testing. After receiving the base station signals in the environment at a fixed time, the model solution could be executed to determine the location of the user, so that a smaller number of base stations could be used to accurately position the user. This could be an improvement over the current downside of a large number of iBeacons needing to be deployed in a large complex space such as Taipei Main Station [20].

## Figures and Tables

**Table 1:** Comparisons of characteristics in various positioning technologies [16].

Wireless Position System	Localization Technique	Range	Accuracy
DOLPHIN (RF with Ultrasonic)	ToA, trilateration	Indoor	2 cm
RFID/INS	RSS/INS	Indoor	2 m
UWB	TDoA/ToA, trilateration	15 m	10 cm
RFID/FPM	RSS/INS	Indoor	1.7 m
Land Marc	RSS, triangulation	50 m	1~2 m
GPS	ToA, trilateration	Global	1~5 m
Radar	RSS, triangulation	Indoor	Indoor
Cricket	ToA, trilateration	10 m	2 cm
Active Bats	ToA, trilateration	50 m	9 cm
Active Badge	ToA, trilateration	5 m	7 cm
COMPASS	RSS, triangulation	15 m	1.65 m
WhereNet (RF)	RSS, triangulation	20 m	2~3 m
LiFS	Fingerprinting database	Indoor	9 m
Bluetooth	RSSI fingerprinting/RSSI theoretical propagation model	Indoor	2~5 m

**Table 2:** Individual Fitting Model Equations for the Different Base Stations.

Base Station	Fitting Model Equation
A	$s=f\left(x,y\right)=-0.01175x^{2}-0.01268y^{2}-77.02$
B	$s=f\left(x,y\right)=-0.007802x^{2}+0.009089y^{2}-79.91$
C	$s=f\left(x,y\right)=0.01176x^{2}+0.009245y^{2}-80.9$
D	$s=f\left(x,y\right)=0.008952x^{2}+0.00218y^{2}-81.45$

**Table 3:** Individual Fitting Model Equations for the Same Base Station Using Data Collected from Different Directions.

Direction Faced	Fitting Model Equation
East	$s=f\left(x,y\right)=-0.01175x^{2}-0.01268y^{2}-77.02$
West	$s=f\left(x,y\right)=-0.01139x^{2}-0.01305y^{2}-73.97$
South	$s=f\left(x,y\right)=-0.01411x^{2}-0.009248y^{2}-75.84$
North	$s=f\left(x,y\right)=-0.0114x^{2}-0.03213y^{2}-70.71$

**Table 4:** Models and Equations Generated from the Four Directions in the Experimental Environment for Base Station A.

	Planar Model and Equation	Fitting Model and Equation
East	s=−0.3888x−0.1935y−74.29	$s=-0.01175x^{2}-0.01268y^{2}-77.02$
	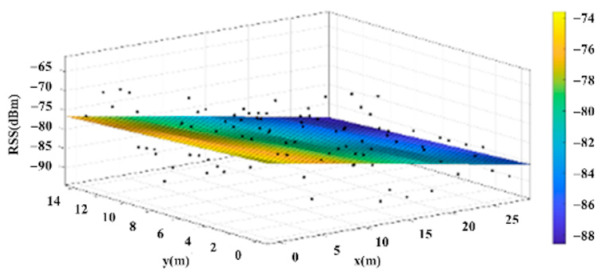	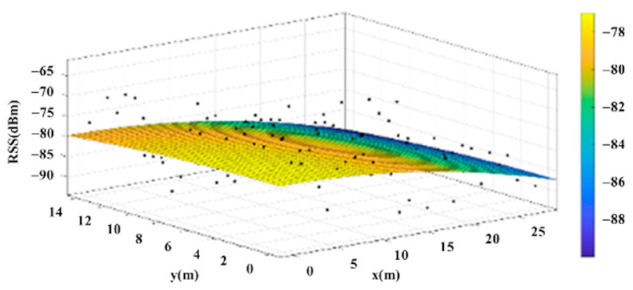
West	s=−0.3418x−0.1775y−71.94	$s=-0.01139x^{2}-0.01305y^{2}-73.97$
	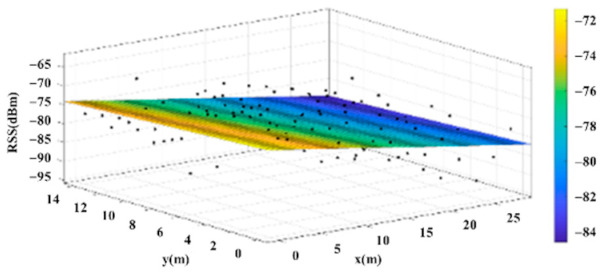	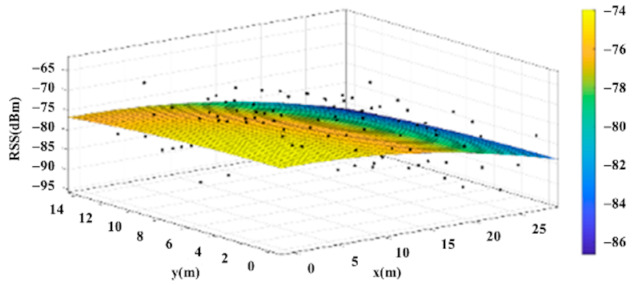
South	s=−0.4371x−0.1187y−73.36	$s=-0.01411x^{2}-0.009248y^{2}-75.84$
	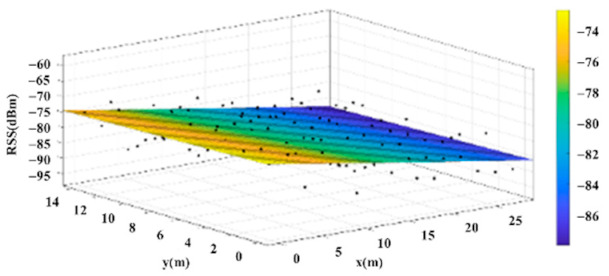	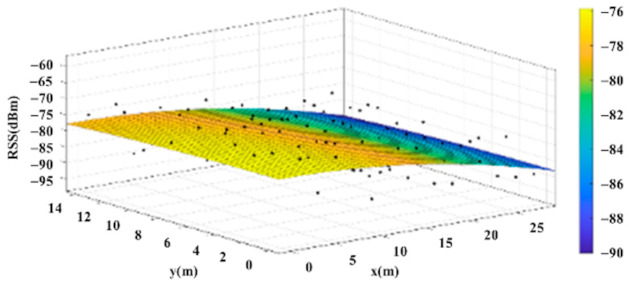
North	s=−0.3678x−0.4942y−67.44	$s=-0.0114x^{2}-0.03213y^{2}-70.71$
	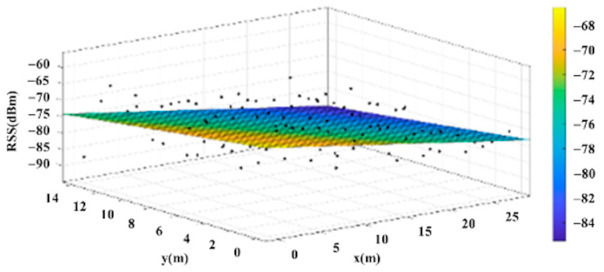	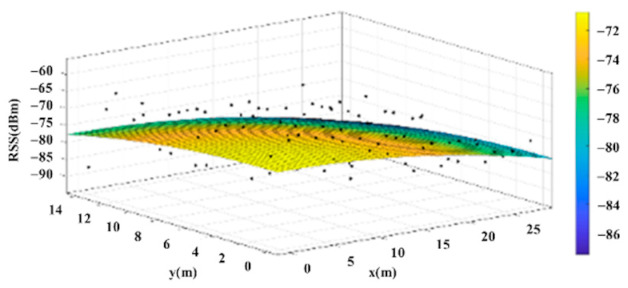

**Table 5:** Models and Equations Generated from the Four Directions in the Experimental Environment for Base Station B.

	Planar Model and Equation	Fitting Model and Equation
East	s=−0.2374x+0.1595y−79.18	$s=-0.007802x^{2}+0.009089y^{2}-79.91$
	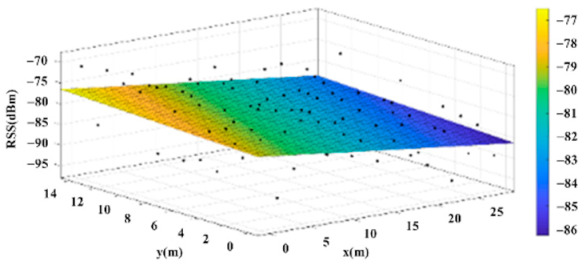	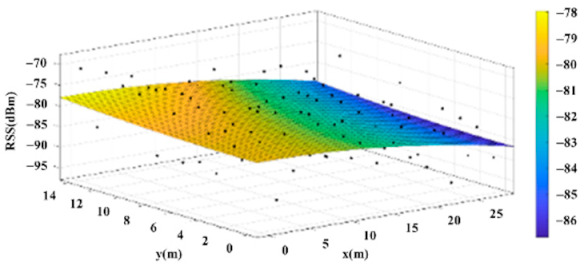
West	s=−0.3475x+0.002143y−73.26	$s=-0.01062x^{2}+0y^{2}-75.23$
	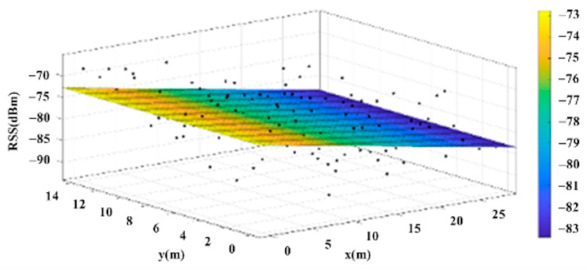	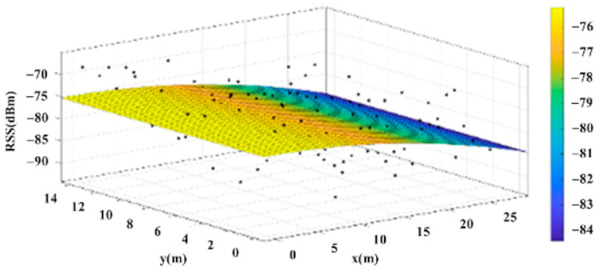
South	s=−0.5678x+0.1538y−70.02	$s=-0.01772x^{2}+0.006322y^{2}-72.54$
	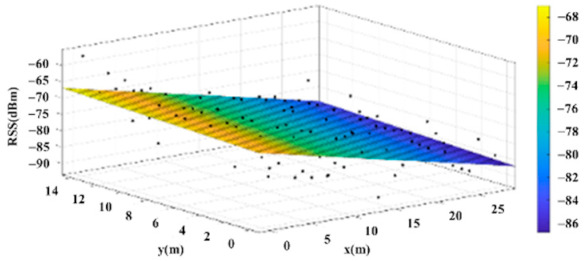	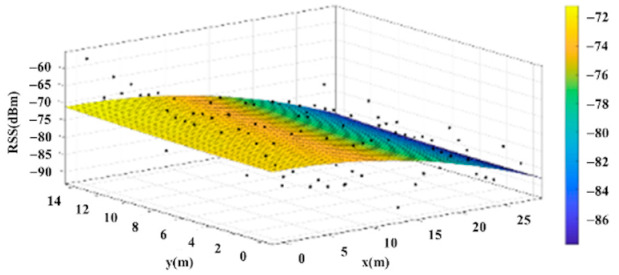
North	s=−0.367x+0.5903y−76.58	$s=-0.01102x^{2}+0.03956y^{2}-77.38$
	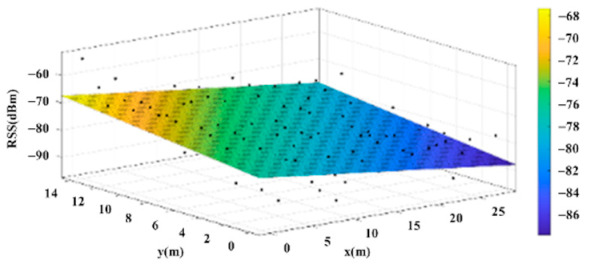	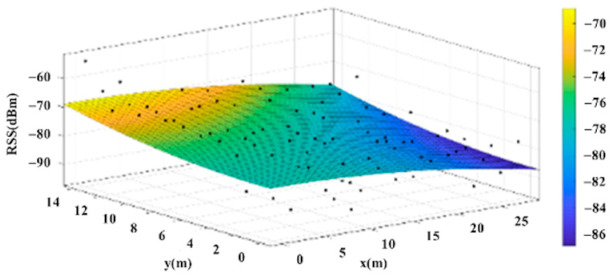

**Table 6:** Models and Equations Generated from the Four Directions in the Experimental Environment for Base Station C.

	Planar Model and Equation	Fitting Model and Equation
East	s=0.3368x+0.1355y−82.74	$s=0.01176x^{2}+0.009245y^{2}-80.9$
	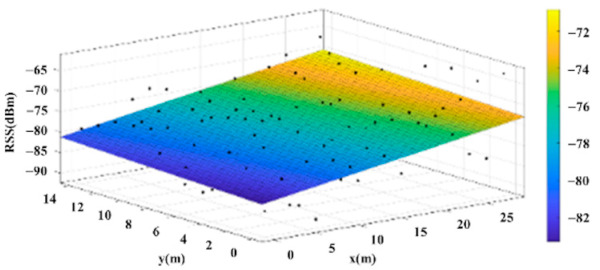	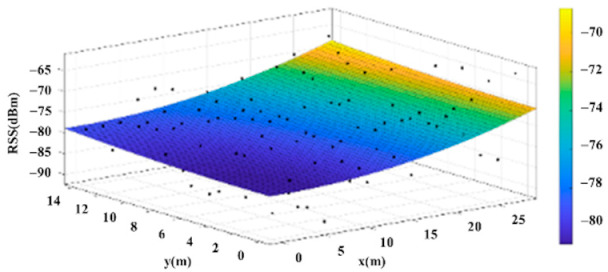
West	s=0.3664x−0.1108y−84.79	$s=0.01197x^{2}-0.007782y^{2}-83.13$
	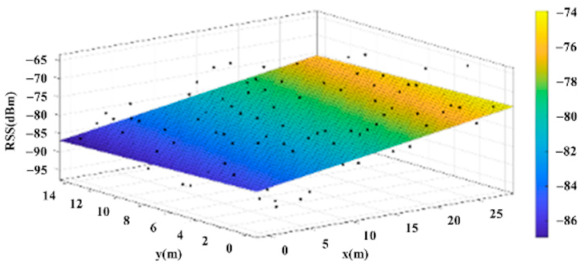	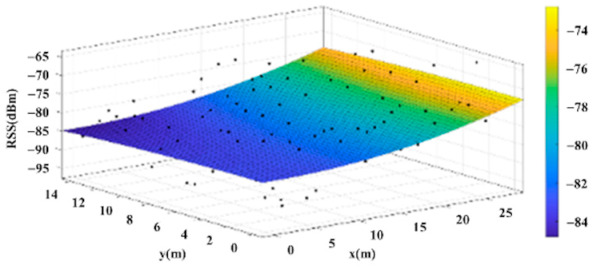
South	s=0.3602x−0.3704y−79.25	$s=0.01272x^{2}-0.02044y^{2}-78.81$
	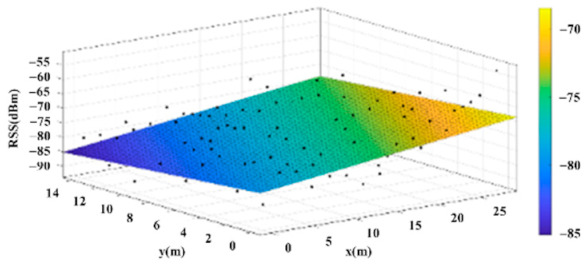	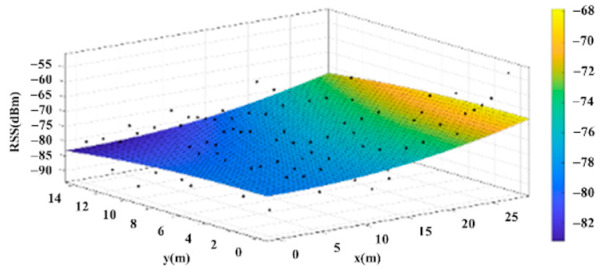
North	s=0.4804x+0.04798y−82.61	$s=0.01668x^{2}-0.0004788y^{2}-80.03$
	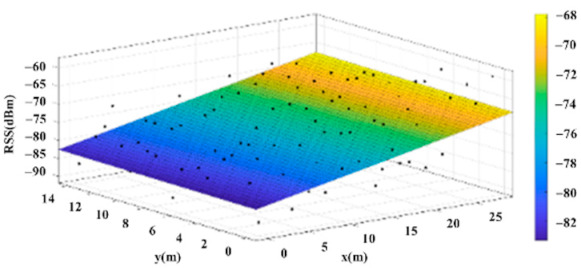	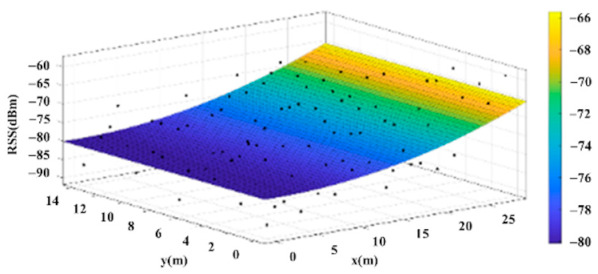

**Table 7:** Models and Equations Generated from the Four Directions in the Experimental Environment for Base Station D.

	Planar Model and Equation	Fitting Model and Equation
East	s=0.2999x+0.05238y−83.44	$s=0.008952x^{2}+0.00218y^{2}-81.45$
	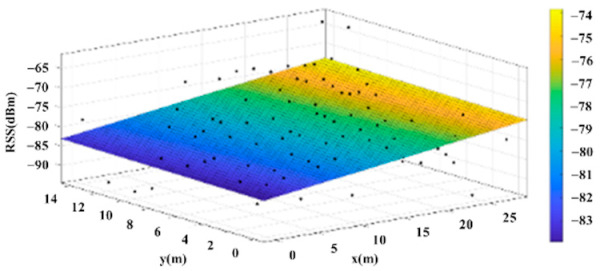	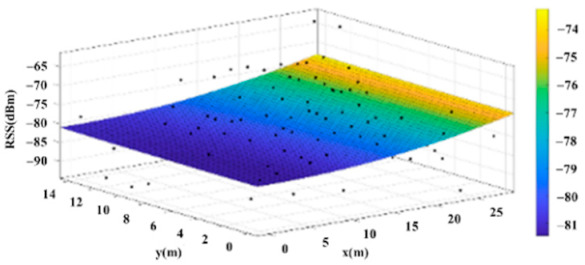
West	s=0.4071x+0.3622y−88.17	$s=0.01393x^{2}+0.02336y^{2}-85.34$
	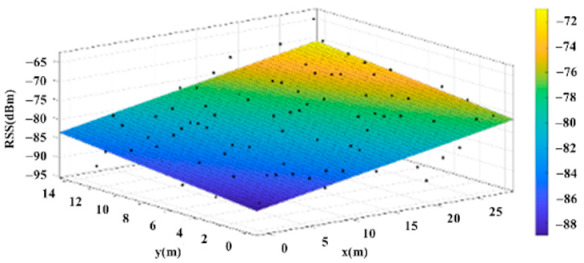	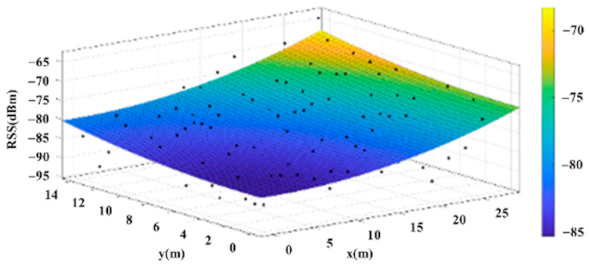
South	s=0.361x+0.3386y−82.61	$s=0.01382x^{2}+0.02518y^{2}-80.69$
	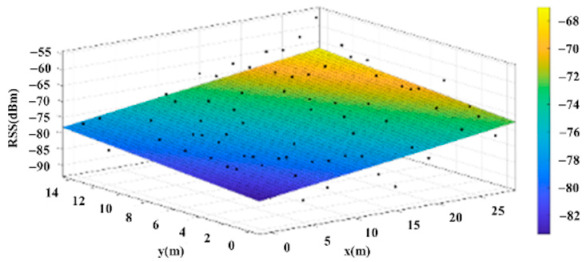	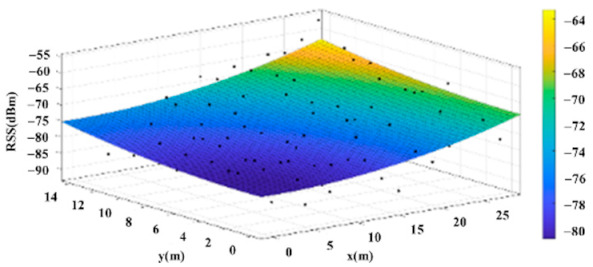
North	s=0.3209x+0.1229y−85.41	$s=0.01058x^{2}+0.01095y^{2}-83.69$
	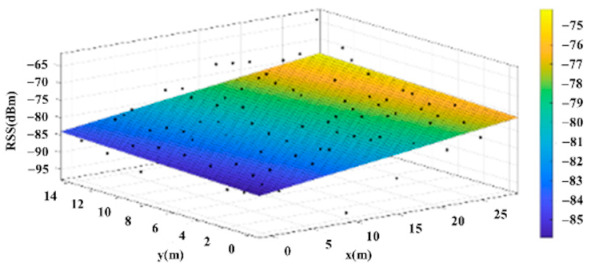	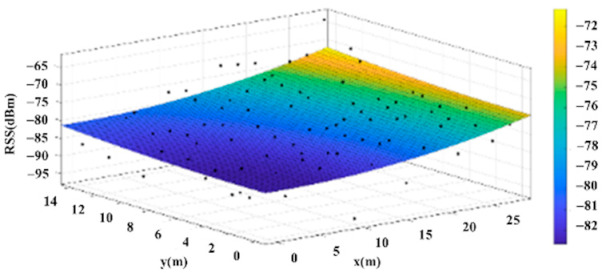

**Table 8:** Comparison of Average Positioning Error on the Basketball Court.

Method	Original	Area	Optimal
Signal Pattern Matching	7.77	6.63	3.56
Planar Model	14.15	8.15	4.34
Fitting Model	9.7	7.82	4.02

**Table 9:** Improvement by Fitting Model Compared to Planar Model Ratios.

Environment	Original	Area	Optimal
Basketball Court (large environment)	31%	4%	7%

**Table 10:** Average Positioning Error Distance for Different Number of Reference Points.

Method (No. Reference Points, Spacing)	Original	Area
Planar Model (120, 2 m)	14.15	8.15
Planar Model (32, 4 m)	13.15	7.88
Planar Model (8, 8 m)	13.12	7.11
Fitting Model (120, 2 m)	9.7	7.82
Fitting Model (32, 4 m)	9.5	7.4
Fitting Model (8, 8 m)	11.15	6.66
